# Spatial memory obviates following behaviour in an information centre of wild fruit bats

**DOI:** 10.1098/rstb.2024.0060

**Published:** 2024-09-04

**Authors:** Emmanuel Lourie, Tomer Shamay, Sivan Toledo, Ran Nathan

**Affiliations:** ^1^ Movement Ecology Laboratory, Department of Ecology, Evolution and Behavior, Alexander Silberman Institute of Life Sciences, Faculty of Science, The Hebrew University of Jerusalem Israel, Jerusalem, Israel; ^2^ Blavatnik School of Computer Science, Tel-Aviv University, Tel Aviv, Israel

**Keywords:** information centre hypothesis (ICH), spatial memory, field manipulation, reverse-GPS, *Rousettus aegyptiacus*

## Abstract

According to the information centre hypothesis (ICH), colonial species use social information in roosts to locate ephemeral resources. Validating the ICH necessitates showing that uninformed individuals follow informed ones to the new resource. However, following behaviour may not be essential when individuals have a good memory of the resources’ locations. For instance, Egyptian fruit bats forage on spatially predictable trees, but some bear fruit at unpredictable times. These circumstances suggest an alternative ICH pathway in which bats learn when fruits emerge from social cues in the roost but then use spatial memory to locate them without following conspecifics. Here, using an unique field manipulation and high-frequency tracking data, we test for this alternative pathway: we introduced bats smeared with the fruit odour of the unpredictably fruiting *Ficus sycomorus* trees to the roost, when they bore no fruits, and then tracked the movement of conspecifics exposed to the manipulated social cue. As predicted, bats visited the *F. sycomorus* trees with significantly higher probabilities than during routine foraging trips (of >200 bats). Our results show how the integration of spatial memory and social cues leads to efficient resource tracking and highlight the value of using large movement datasets and field experiments in behavioural ecology.

This article is part of the theme issue ‘The spatial–social interface: a theoretical and empirical integration’.

## 1. Introduction

To reduce uncertainty surrounding the dynamic availability of resources in their environment, animals must continuously process and adaptively respond to relevant information inputs. Generally, individuals have two sources of information at their disposal: self-acquired experience or memory, and information acquired socially by interacting with, and monitoring the behaviour of, conspecifics [1–4]. The degree of investment in acquiring social information is contingent upon the species’ social structure and resource distribution dynamics. It has even been suggested that the evolution of group living, ranging from fission–fusion societies to highly integrated group foraging, has been shaped by the benefits of using social information to predict resource availability in space and time [5–8]. This is ultimately because less-predictable resources usually select for greater reliance on social information to facilitate finding them, whereas exploitation of more predictable ones favours memory use [9]. Yet, some resources may be predictable spatially, but unpredictable in the temporal emergence cycles, leading individuals to integrate personal and social information acquisition strategies [10–12]. For example, plants are spatially predictable, making it relatively straightforward to memorize their locations and find them independently. Yet, their temporal phenological dynamics—such as the emergence of leaves, flowers or fruits—often vary substantially within and among species and regions, making the complementary use of social information advantageous to determine when to visit them [8,13].

Multiple mechanisms allow conspecifics to learn from each other about unpredictable food resources (for a detailed review, see [8]). Many species use local enhancement at small spatial scales to detect foraging hotspots by monitoring the activity of conspecifics *en route*. Famous examples include marine birds scanning for congregations of plunge-diving birds or group-foraging of insectivorous bats eavesdropping on the feeding buzzes of their neighbours [14,15]. At larger scales, foragers that congregate at roosts, leks or breeding colonies can use these locales to share information about new food discoveries. According to the information centre hypothesis (ICH; [16]), new information is introduced by successful foragers returning to the congregation site, where it can be received and evaluated by naïve conspecifics, who then follow the informed individuals to the location of the new resource.

Since its proposition, debate on the ICH has led to the refinement of the evolutionary theory behind the development of information centres and the mechanisms through which they operate and persist [17–19]. For one, the ICH denotes that information-sharing is the key driver of the evolution of communal roosting or breeding aggregations. Yet, there are multiple additional advantages to communal roosting that are unrelated to information (such as thermoregulation, e.g. [18,20,21]), or advantages of sharing non-food information (e.g. about breeding opportunities and predator defence [22,23]). Importantly, the ICH discusses these two aspects in conjunction, while the approaches and methodologies to examining the evolutionary drivers of communal roosting (e.g. evolutionary models) differ substantially from examining if and how an information centre operates (e.g. field experiments).

In addition, the ICH assumes information is selectively shared at the congregation site for potential beneficiaries to perceive and (eventually) reciprocate [18,19]. However, returning foragers probably advertise information that is impossible to hide within clusters of individuals, such as fatness, enlarged crops, visible food items or familiar scent cues [18]. In such cases, the information is public, and sharing it can be considered a mild form of information parasitism [1,18,19,24].

Fifty years after the ICH proposition, convincing empirical evidence for it in wild animals is scant, mainly owing to the challenges of testing it in the field [17,19]. As Mock *et al*. [17] outlined, one essential prerequisite for the ICH is to show that uninformed individuals physically follow informed ones to the new source, i.e. following behaviour. However, these instances could be rare and thus technically challenging to document without highly detailed tracking data of many individuals from the same colony and corresponding information about the spatiotemporal dynamics of their resources [8,25–27]. This is perhaps why many studies revert to different metrics, such as departure synchrony from the roost with similar bearings, making the assumption that co-departing conspecifics follow each other as well [28–31]. Yet, departure synchrony could be explained by non-social factors, such as diel and climatic conditions, or leaving the colony together to dilute predation risk [18]. More recently, Harel *et al*. [27] provided more direct evidence for following behaviour in the wild by documenting how uninformed Eurasian griffon vultures followed informed vultures the long way from the nest to the carcass, in frequencies exceeding those of uninformed dyads.

There are common features to these and other studies of the ICH (e.g. [28,32,33]), which may unjustifiably treat the observation of physical following as a prerequisite for the use of information centres. These studies, and the ICH’s premise, are on species that forage and acquire information about highly patchy and ephemeral resources [16,19]. Yet, information centres were also shown in other systems, most famously in eusocial honeybees [34], which routinely forage on spatially predictable flowers and still use the hive as an information centre to find new resources [34–37]. Interestingly, honeybees probably do not follow information-carrying hive-mates: in feeder experiments, translocated honeybees that were exposed to the illustrious ‘waggle dance’ (which was shown to transmit information on the direction and distance of resources [36]) flew to the vicinity of where the feeder should have been, suggesting they could not have followed other honeybees, nor used additional landmarks, to get there [35,36,38].

Indeed, although the honeybees’ ‘dance language’ delivers incredibly detailed information about the flowers’ location, the lure in the sophistication of it may have overshadowed two additional findings, suggesting the dance is used primarily to supplement the knowledge honeybees already have. For example, most dance-observing honeybees (approx. 80%) did not proceed to look for an entirely new flower, but instead probably used the social information to confirm personal memory of resources they visited in the past [39]. Additionally, the waggle dance may not be the main pathway through which hive-mates share information. Even in von Frisch’s canonical study [34], the floral scent introduced to the hive prompted honeybees to fly in the right direction, without the waggle dance. Additional experiments support these alternative pathways by showing that nectar-scented plumes artificially blown into the hive triggered the recruitment of honeybees to the right flower direction [40] and that honeybees can re-use this information up to 4 days after being exposed [41]. This, together with similar examples in ants [42] and wasps [43,44], showing they can match odours to memories of their locations, also known as ‘associative recall’, strengthens the notion that simple and public cues, together with spatial memory, may suffice for naive individuals to navigate towards the newly emerging resource. Surprisingly, the question of whether non-eusocial and diploid species—particularly social vertebrates with more developed spatial memory—similarly integrate spatial memory and simple social cues to find new resources, remains uninvestigated [17,45].

Another underinvestigated option is that there may be additional locations outside the roost (or hive) where individuals can exchange information. For example, some marine birds, namely Guanay cormorants and Australasian gannets, congregate at sea to form ‘compass rafts’ near the breeding colony, which they use to monitor incoming successful foragers [31,46]. Moreover, birds leaving together from rafts tend to depart more synchronously than birds from nests, suggesting they may follow each other when departing from rafts [31]. Similar to marine birds, it is possible that other species exchange information at foraging hotspots (e.g. [47]), using them as alternative or supplementary sources of information [48].

In this study, we test for an alternative information transfer pathway at an information centre that emerges from integrating social information and spatial memory, obviating following behaviour. To this end, we conducted field manipulations and studied the movement patterns of more than 200 Egyptian fruit bats (*Rousettus aegyptiacus*), which we tracked at high spatiotemporal resolution using the Advanced Tracking and Localization of Animals in Real-life Systems (ATLAS) reverse-GPS system in the Hula Valley, Israel [49–51]. Note that the manipulations test whether information is shared in the communal roost, but do not aim to assess additional, mostly controversial, statements made by ICH regarding the role of information transfer in driving the evolution of communal roosting and breeding locations [18].

**Figure 1.**
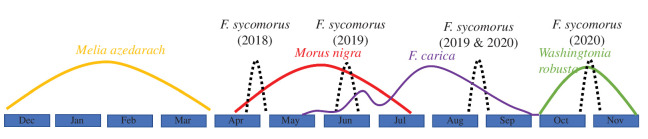
Schematic representation showing the observed fruiting cycles of the main tree species visited by Egyptian fruit bats in the Hula Valley, Israel. Curves signify the average proportion of ripe fruits of each species in the study area. For example, the average number of ripe fruits observed on the only two *Ficus sycamorus* trees in the study area, were asynchronous across years (2018–2020), hence considered unpredictable to bats. All other illustrated fruit tree species are seasonal and longer lasting, making them more temporally predictable.

Several reasons make this species appropriate for examining this pathway. Firstly, fruit trees are spatially predictable, but some favourable species (in our study system) are temporally unpredictable (figure 1). Accordingly, bats are expected to use a mix of personal and social information acquisition strategies. Secondly, repeated and large congregations in cave-roosts and around fruit trees, together with a developed olfactory system, suggest conspecifics can detect odour cues of a new incoming fruit (52,53). Additionally, evidence from a similar species—the Peters’ tent-making fruit bat—demonstrates they are attracted to social information about food, based on the finding that they preferentially interact with roost-mates that carry new food odours [54,55]. Lastly, previous research demonstrates unique and advanced spatial memory in *R. aegyptiacus*, to the extent of obtaining a ‘cognitive map’ with information about the locations of all relevant trees in the landscape [50,56]. Strong reliance on spatial memory in this species is probably the reason why two extensive tracking studies report they most probably never follow each other from the roost (approx. 0.3% tracked bats [6], or during other foraging movements (0.4% of paired flights tracked [50]). Concurrently, our main hypothesis is that while bats acquire information from conspecifics about when a resource is available, they rely on their spatial memory to know where to find it.

To test this hypothesis, we experimentally manipulated the introduction of new information to the roost by releasing bats smeared with the fruit pulp of the temporally unpredictable *Ficus sycomorus* tree species (figure 1), during the time it bore no fruits, of which there are only two individual trees in the landscape. We then tested the prediction that if learning new resources occurs based on new odour cues in the cave, naive roost-mates that were tagged and already roosting in the cave, will visit the fruitless trees more frequently than expected during routine exploration of these trees when they bore no fruits. Furthermore, we examined the hypothesis that foraging trees serve as additional sources of information, mainly because these bats select a very small proportion of all available trees, which they revisit for many nights continuously [50]. Hence, we predict that encounters around trees will also lead bats to learn from each other and, consequently, visit unfamiliar trees.

## 2. Methods

### (a) Study species and area

The Egyptian fruit bat is a widely distributed Old World fruit bat (Pteropodidae) and the only species found beyond the tropics [49]. In Israel, it is highly abundant, only recently upgraded from a pest to a least-concern protected status in 2020 [57]. The species forages on a large variety of native and exotic fruit tree species [50,58], some of which, especially of the *Ficus* genus (Moraceae), produce fruits at unpredictable, non-synchronous, timings (e.g. [59,60]). Every foraging night, bats feed on a small subset of the available trees and repeatedly revisit them for weeks [50], a typical behaviour for species that forage mostly on predictable and long-lasting resources and, therefore, rely heavily on memory [6,12,53]. It is common to observe bats arriving at fleshy fruit trees immediately when they ripen, and the question remains how do they locate them so quickly?

The Hula Valley in northern Israel is a 19 000 ha mosaic of agricultural land and small settlements characterized by an abundance of fruit trees and orchards [50,53]. We have mapped almost all fruit trees *n* = 13 477) and orchards (*n* ≈ 18 000 trees) in the study area, among which the bats use primarily seasonal trees and approximately 7% temporally unpredictable species (figure 1; [50]). Among these, the most frequently visited unpredictable species belong to the *Ficus* genus, namely, *Ficus religiosa (n =* 8 trees*), F. sycomorus (n* = 2 trees), and *Ficus microcarpa (n* = 12 trees). The valley inhibits a moderately sized population of approximatley 2500 bats that forages almost exclusively within it and repeatedly returns to the same 1–5 cave-roosts containing up to approximately 1400 individuals [53]. Roosts were found to be composed of non-related individuals [61,62]. In previous work, we found that bats from the two main roosts, Gershom and Zemer, are highly faithful to them and that bats from each roost use segregated foraging areas with a small overlap of trees, a partitioning that is probably driven by memory and information transfer within roosts [53].

### (b) Captures and tracking

Overall, 233 bats were captured using mist nets, either around trees or at cave exits, and their weight, forearm length, sex and age (categorical) measurements were recorded [63]. Bats were tracked during 2018–2022 using the ATLAS [64], Localization of Animals in Real-life Systems [64] reverse-GPS system at 0.5–0.125 Hz sampling rate and with a 5 m median localization error [51,65]. For adults, tags were mounted using a shrink-coated bead-necklace collar with a weak link to allow spontaneous drop-off after less than 1 year, giving up to nine months of tracking. For sub-adults and juveniles, tags were glued on their upper backs with surgical cement (Perma-Type, Plainville, CT, United States), lasting about three weeks. Tag units weigh 7.4 g (5.2% of mean adult body mass) for collars or 3 g without. For more details on the ATLAS system and tag performance please refer to Toledo *et al*. [50] and [66]. Captures and tagging procedures were approved by the Ethics Committee of the Hebrew University (NS−21−16656−3) and the Israeli Nature & Parks Authority (yearly permits 2020/42577, 2021/42850, 2022/43098).

### (c) Filtering and segmentation of movement tracks

For the analysis of routine movement tracks (routine visits to the experimental trees and tree encounters), we first filtered raw bat tracks using quality filters for localization errors based on the covariance matrices attributed to each ATLAS fix and a speed filter of 20 m s^−1^ (for more details on pre-processing, see Gupte *et al*. [51]). Then, we segmented the tracks into phases of tree visits and commuting flights (between trees/roosts). Identification of tree visits used a first-passage algorithm to determine the centre of a ‘cloud of fixes’ where the animal has spent a specified number of observations within a certain radius (github.com/ATLAS-HUJI/R/tree/master/AdpFixedPoint). Each tree visit was annotated with its coordinates, visit duration and tree species derived from a tree dataset collected in previous studies [50,53]. All analyses were conducted in R (v. 4.2.2 [67]).

### (d) Field manipulations

**Figure 2.**
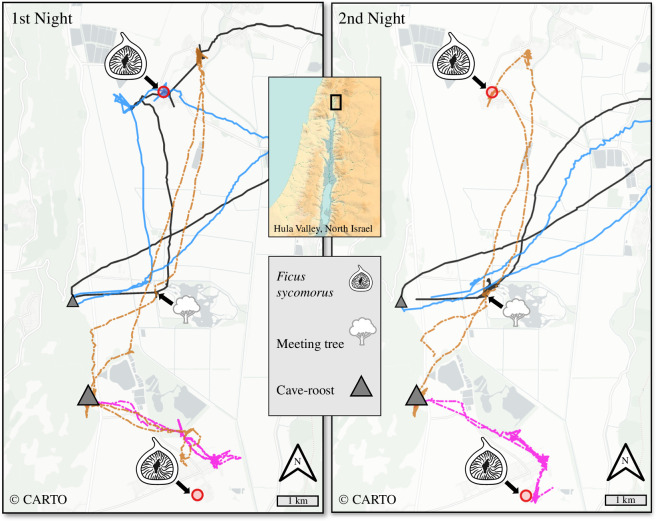
Tracks of four Egyptian fruit bats leaving the main cave-roosts (grey triangles), two from Gershom (dashed tracks) and two from Zemer (solid tracks). Panels show the first night (left) and the second night (right) after the experimental manipulation introducing bats smeared with the pulp of *F. sycomorus* to the roosts. The only two *F. sycomorus* trees in the study area (red circles and a fig icon), bore no fruits more than 2 weeks before the experiment. On the first night, one smeared bat (black tracks) and one naive bat (blue) from Zemer visited the northern *F. sycomorus* tree, while the ones roosting in Gershom did not visit any. The second night, one bat from Gershom (orange) also visited the northern fruitless tree, which could be in reaction to the odour of a smeared bat it roosted with in Gershom (not illustrated) or to another smeared bat (black) it met on a tree (tree icon). See figure 3 for details on the relative number of bats visiting the focal trees during the manipulation and routine movement.

In the field manipulations, we focused on the *F. sycomorus* species, as it is a large, conspicuous tree with temporally unpredictable fruiting (figure 1; [59,68]). Only two individual trees are found in the entire landscape (figure 2; 33.05° N, 35.60° E, and 33.16° N, 35.59° E), making the identification of true visits to it more straightforward and particularly relevant for unpredictable resources. Manipulations were carried out in three campaigns, during June, July and December 2020, when different tree species produce fruits (figure 1). Prior to the manipulations, we tagged and tracked the movements of a total of 56 bats for more than two weeks before the manipulation and ensured none visited any of the target trees, and that the trees bore no fruits during the preceding two weeks. We also verified that none of the bats, including untagged ones, arrived at the target trees three and one night before the manipulation by observing the focal trees directly.

**Figure 3.**
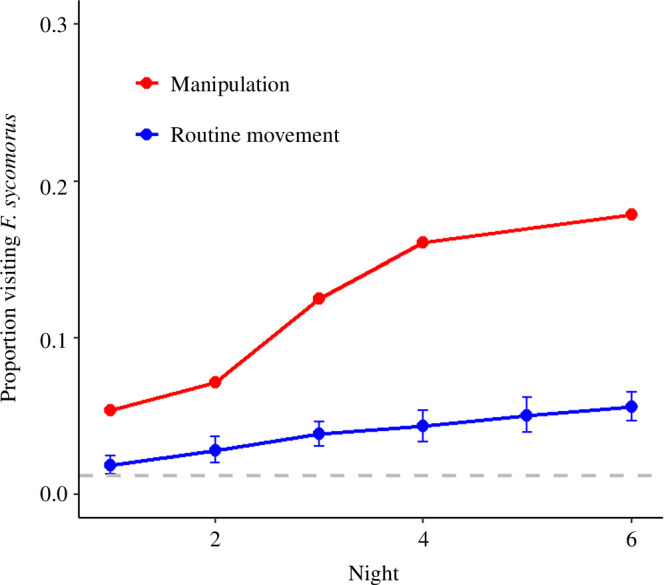
Proportion of naive bats visiting any of the two *F. sycomorus* trees 1–6 nights after the manipulation introducing odour-carrying bats to the cave-roosts (red), and for the same night-intervals for tagged bats during routine movements when the focal trees bore no fruits (blue). The grey horizontal line denotes the proportion of bats that visited the focal trees two weeks before the manipulation (=0.012), among those included in the manipulation (*n* = 71, manipulated and naive). For all time intervals, the proportions of the manipulation were significantly higher than the ones of the routine movements (*p* < 0.01, permutation test). Blue whiskers denote the confidence intervals around the mean proportions derived from 200 to 230 randomly sampled time intervals (without replacement).

On each of the three manipulation nights, we captured a total of 16 foraging bats around trees and kept them in a soft cage (in groups of 2–5 individuals) under supervision in a dark room at the Hula Research Center (33.11° N, 35.58° E). To prevent further foraging activities, bats were released at the roosting cave shortly before sunrise, after being fed and smeared with the fresh or defrosted pulp of *F. sycomorus* figs we had collected and blended from other trees across the country several days prior. These 16 bats were smeared thoroughly on their torso, and not on their wings, to create a very strong odour-cue, mimicking the natural (but probably slighter) fruit pulp scent covering their bodies while foraging (e.g. from other bats’ secretions, while searching for fruits in the tree canopy), and we assume this made the new odour last for a long time on the manipulated bats for several nights after their release. Additionally, smeared bats voluntarily fed from the fruit pulp, so the scent was also carried in their mouths. We then released the smeared bats approximately 5 m outside the cave, to avoid disturbance at the roost. Smeared bats were released in the two largest roosts, Gershom (up to 1300 bats) and Zemmer (up to 700 bats), in conjunction and were also tagged. All bats entered the cave and roosted there for at least the rest of the day.

Out of the 233 bats considered in this study, 72 were tagged during the manipulations, of which 16 were smeared and 56 were naive (table 1). To estimate the proportion of bats visiting the focal trees, we counted the number of naive bats visiting the *F. sycomorus* trees and those that did not during the following 1–6 nights, assuming the bats could memorize or re-smell the cue during these intervals but not beyond this period.

**Table 1.** Summary of the manipulation results of bats visiting the *F. sycomorus* trees up to six nights after the manipulations during three campaigns in 2020. (The table includes counts of the bats smeared with the trees’ fruit pulp and released in each of the two main cave-roosts (i.e. manipulated bats), the date of the introduction and the number of naïve tagged bats roosting there at the same time. The two last columns are the sum of all manipulated bats (*n* total) and the number of bats visiting any of the two *F. sycomorus* trees during the manipulation period, separated into the number of manipulated and naive bats. In bold is the sum of all bats included in the experiments and the number of naive bats visiting the trees, from which the proportion of visiting bats in the experiment (here, after six nights) was calculated.)

date	cave-roost	*n* manipulated	*n* naive	*n* total	*n* bats visited *F. sycomorus* (naive | manipulated)
22 June 2020	Gershom	3	9	12	4 | 2
22 June 2020	Zemer	4	6	10	2 | 1
7 December 2020	Gershom	4	15	19	2 | 1
7 December 2020	Zemer	1	7	8	1 | 0
19 July 2020	Gershom	4	19	23	1 | 0
19 July 2020	Zemer	0	0	0	0 | 0
sum (after six nights)		16	56	72	10 | 4

### (e) Comparing manipulation results to routine movement during non-fruit periods

To assess whether the manipulation led to increased visitation to the *F. sycomorus* trees, we compared the proportions of naive bats visiting the trees during the manipulation with those of all tracked bats during non-fruit periods. The manipulation period’s visitation proportion was defined as the accumulative number of bats visiting the *F. sycomorus* trees 1–6 nights after the manipulations, divided by the total number of naive bats tested, across the three campaigns (*n* = 56).

For the probabilities of visiting the trees during routine movements, we first collected the tree visitation patterns of all bats during non-fruit periods from 2019 to 2023, based on phenological surveys of the *F. sycomorus* trees. Then, we randomly sampled all observed 1–6 nights time intervals, and calculated the proportions of bats visiting the trees relative to the number of all tagged bats. For comparability with the manipulation conditions, we aggregated, for each time-interval iteration, these random samples to include at least 70 bats overall, mimicking the manipulation’s sample size. This often required combining multiple intervals to reach the necessary bat count. For example, if a single six-night interval yielded fewer than 70 bats, we would draw another six-night interval and cumulate the visit counts until reaching or exceeding the 70 bats’ threshold. This process was repeated for each interval, resulting in 200–230 observed proportions per interval. In addition, we also compared the manipulation results against the proportion of the naive bats that visited the focal trees two weeks before the manipulation.

Finally, we tested the statistical difference between the proportions of visits to the focal trees in the manipulation and those taken from 200 to 230 randomly sampled iterations from the routine movements. The *p*-values were estimated as the number of instances where the observed proportions from the manipulation exceeded the proportion from the randomly sampled routine movements (a one-tailed test).

### (f) Analysis of information transfer on trees

All tracked bats, but one, were found at a given time and tree with at least one other tracked bat. We consider these instances as interactions between bats that allow for information transfer through odour detection. To estimate the potential use of trees as additional, secondary, sources of information, we analysed the probability of a focal bat visiting any tree already visited by the bat it met but which was not yet visited by the focal bat (i.e. a new tree). Specifically, for each tree encounter, we recorded the unique trees visited by a bat in the last three nights before the meeting. The focal bat’s new trees were defined as the ones it visited during the subsequent three nights, excluding those it already visited. Then, for each bat after a meeting, we determined whether its new trees overlapped with the already-visited trees of the bat it met.

As a control group, we randomly selected an equal number of tracked bats that did not meet, from the same months as the bats that have met and conducted the same analysis for these individuals. We then pooled both datasets and created a logistic regression model with a response indicating whether bats used each other’s trees (a Boolean 1/0 response variable) and if the dyads met or not as the predictor variable. To account for the possibility that bats can use the acquired information with a delay, we ran separate models for time lags of 1–6 nights after the meeting event. We also repeated the same analysis but focused only on new unpredictable species (*Ficus* genus), to account for the possibility that the motivation to learn about unpredictable species could be higher than for more predictable tree species.

## 3. Results

### (a) Field manipulations support the information centre hypothesis in the communal roost

Out of the 56 naive bats included in the experiment, 10 visited one of the two fruitless trees in the study area (approx. 18%; figures 2 and 3). Note these do not comprise the manipulated bats that were smeared with *F. sycomorus* odour, of which 4 out of 16 visited one of the trees. We defined a ‘visit’ to the fruitless trees as any instance where a bat circled around a tree, even briefly, excluding instances where it flew straight over the tree. The average duration of these visits was 9 min, ranging from 4 s to 50 min. The time between initial exposure to the tree’s visits varied among bats with three naive bats visiting the trees the first night after the manipulation, four 2–3 nights after, two after four nights and one bat visiting the tree six nights after. All naive bats that visited the *F. sycomorus* trees foraged on other tree species before, with an average of two trees (of any species) visited prior (range: 0–5).

Two findings suggest these visits were a response to the manipulation, exceeding the visitation rate to these trees during routine exploration flights. First, the proportion of visits to these trees made by all tracked bats two weeks before the manipulations were close to zero (0.012; figure 3; electronic supplementary material, figure S1). Second, the proportion of bats visiting the trees during the manipulation was significantly higher than that of tracked bats during routine movements when the trees bore no fruit, observed across all tracking periods, derived from sampling at random 200–230 iterations of the same night intervals as in the manipulation (figure 3; electronic supplementary material, figure S1).

### (b) Little support for information transfer on trees

Bats encountered each other frequently (*n* = 233), based on the relative frequency of instances they visited the same tree at the same time. Among all dyads tracked simultaneously over a six-night period, 31% were observed sharing a tree at least once, a proportion that was higher for same-colony members (42%) compared to bats from different cave-roosts (25%).

**Figure 4.**
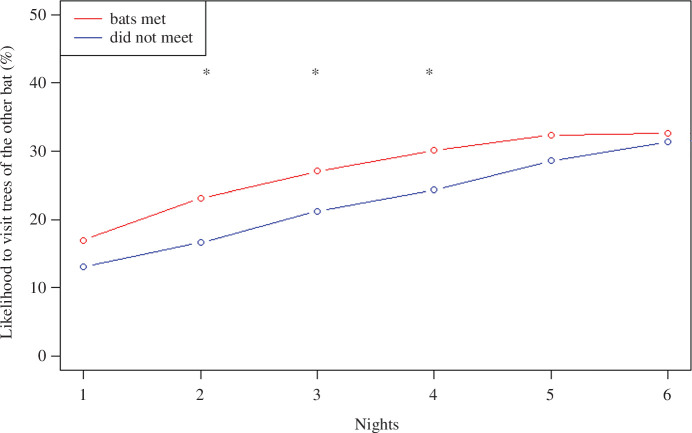
Results of encounters on trees, comparing the likelihood of a bat discovering new trees that were visited by another bat it met on a tree at night = 0 (red lines), against an equal number of dyads that did not meet (blue lines). Dyads that met on trees were only slightly likelier to visit new trees of the other bat (<6%), and only significantly so during the 2–4 nights after the encounter (*p < 0.05). Results are derived from multiple logistic regression models (one per night after the meeting) and suggest minor evidence for trees serving as information centres.

To elucidate whether bats that met on trees proceed to learn about new tree resources, we estimated the probability of discovering new trees that the bat it potentially met already visited, and compared the mean probabilities of dyads that met, against the ones of randomly chosen dyads that did not meet during the same periods (figure 4; electronic supplementary material, figure S2). We found significant differences in the probabilities of bats that met on trees against dyads that did not, for two, three and four nights after they met. However, because these differences were small (<6%; figure 4), we interpret them as little support for the exchange of information on trees. Similar relationships were found when sub-setting the new trees to include only unpredictable species, with no significant differences between groups (electronic supplementary material, figure S2). Anecdotally, however, we observed two bats that visited the target tree which may have done so following a meeting with an odour-carrying bat on a tree, before or without encountering it in the communal roost (see example in figure 2).

## 4. Discussion

### (a) Spatial memory obviates following behaviour in the bats’ information centre

In this study, we used both experiments and highly detailed movement data to elucidate an alternative pathway for the ICH. Specifically, we demonstrated the ICH in the understudied, yet widespread, circumstance of foraging on resources that are spatially predictable but fruit unpredictably (1–8 cycles a year [59]), and asynchronously (days to months, in our study system; e.g. figure 1). Our findings suggest that inadvertent social information, in the form of odour-cues carried by incoming roost mates, drove bats to visit the corresponding new resources using spatial memory, obviating the need to follow informed individuals. To our knowledge, such a pathway of information transfer from an information centre was only documented previously in colonies of eusocial insects [4,36,40,42,44].

These inferences are drawn from the results of an experimental manipulation, a complementary analysis of movement patterns derived from 233 bats, and previous research highlighting the existence of excellent spatial memory and showing no support for following behaviour in this species [6,50,53]. The existence of only two unpredictably fruiting *F. sycomorus* trees in our study site (figure 2) enabled us to conduct an experimental manipulation rarely applied in previous tests of the ICH in the wild [69]. Specifically, we found that odour-carrying bats introduced to the cave drove naive individuals to visit the fruitless trees 1–6 nights post-exposure, with probabilities exceeding those of routine visits to the same (fruitless) trees, and the ones of the same naive bats tracked two weeks before the manipulation (figure 3; electronic supplementary material, figure S1). In contrast to roosts, we found weak support for congregations on trees functioning as additional information transmission sites, since encounters with conspecifics on trees did not substantially increase the number of visits to new trees (figure 4; electronic supplementary material, figure S2).

It is commonly claimed that foragers use social information when its benefits outweigh personal knowledge [9,70]. Here, we show how bats integrate both information strategies to discover newly emerging fruits, by monitoring social cues to know when resources emerge, and their self-acquired spatial memory to pinpoint their locations. While such integration was theorized before (e.g. [13,71]), most empirical examples were documented in honeybees. For example, Reinhard *et al*. [40] performed a similar experimental manipulation to this study by blowing flower-scented odour-plumes into the hive and found that naive honeybees were recruited to the corresponding empty (and odourless) feeder. Interestingly, bees found the feeder without following conspecifics or using the waggle dance, a behaviour that was probably facilitated by their evolved spatial memory [35]. Similar examples of the ICH are scarce, despite the diversity of resource predictabilities vertebrates forage upon, and prolific research on the developed cognitive skills of many social birds and mammals [4,11,72].

We cannot, however, dismiss the possibility of following between bats completely [62,73]. Some eusocial species, for instance, follow one another indirectly using trails left by successfully returning foragers, such as ants’ pheromone trails [74] and body-odour trails made by scouting naked mole rats [75]. Similarly, trail-following could theoretically occur in non-eusocial colonies. Using simulation models and data from a radar-tracked common tern colony, Urmy [76] proposed that seabirds use ‘visual trail following’ by aligning their outbound paths with the direction of returning birds (e.g. [14]). Accordingly, uninformed individuals prioritize following inbound traffic of birds carrying prey items, rather than identifying and following specific informed birds leaving the colony [26,76]. Along the same lines, it is possible that *R. aegyptiacus* also follow heavy conspecific traffic, for instance, of many or specific bats leaving the cave in the same direction. Moreover, since other unpredictable species have more than two trees in the landscape (e.g. *F. religiosa*), bats are expected to improve the probability of visiting the right one by following the general direction of informed conspecifics as they depart. Since tracking evidence suggests the opposite, that bats do not follow each other when exiting the cave [6] or en route [50], it is worth noting that higher-than-random observations of non-random tandem flights, which are considered the most reliable ways to show the existence of following behaviour [19,22], especially when rare as in this system, are difficult to observe with standard tracking technologies. This is namely owing to insufficient temporal sampling and the (usually unknown) quantity of untagged individuals involved [27]. We believe the use of Eulerian technologies, such as radar, to be an important complementary tool for such cases, as they can monitor the flight characteristics of all individuals within the radar radius simultaneously [66] and recently proved useful in detecting individual free-ranging bats in the same study area [77]. Lastly, our results also demonstrate that bats tended to visit other known trees before they visited the newly fruiting tree (§3.1). This strategy may support the hypothesis that they do not need to follow each other if the information is only used opportunistically while passing near one of the trees of the newly fruiting species.

### (b) Social information is probably advertised publicly via odour-cues in the roost

An increasing number of studies uphold the more general case of the ICH, whereby public, rather than intentional and reciprocal, information sharing is more evolutionary-stable and widespread across communal species [1,18,24,78]. Among the different food-related cues bats may share, odour is most likely a public signal, as it is a relatively long-lasting cue that rapidly spreads across the entire cave-roost [24]. The potential implementation of associative recall, whereby animals, including humans, associate odours with memories of their locations [79], probably explains our findings that bats visited the tree after the manipulation (figures 2 and 3) without following conspecifics. Accordingly, we believe new odours entering the roost serve as a strong and persistent social cue that delivers public information about the exitance of new resources and can provoke memory of the new foods’ location. This mechanism is especially relevant to fruit bats (Pteropodidae and Phyllostomidae), which have a highly developed olfactory system, were found to prefer to interact with unfamiliar roost-mates when they bring novel fruit odours, and were practically never recorded following each other [50,54,55,79].

To elucidate further whether and how public information is transmitted in the roost, continuous monitoring of roosting behaviour is needed (e.g. as in [61,80]). It would then be possible to test, for example whether bats use vocal communication to learn about new food as well. Indeed, roosting *R. aegyptiacus* vocalizes frequently and exhibits a diverse vocal repertoire [81]. However, based on an exhaustive audio recording dataset, Prat *et al.* [82] found that most communications among roosting bats are agonistic, which is an unlikely way to transfer helpful information. Moreover, calls were directed toward specific individuals, and rarely broadcasted publicly.

### (c) Considering variation in social information use among individuals

An additional interesting finding from our field experiment is that individuals varied in their response to the inadvertent social information—some visited the trees later than others (figure 3; electronic supplementary material, figure S1), and some appear to have not responded to the information whatsoever (57 out of 72,). These individual-level differences may attest to variations in internal traits, such as energetical needs or consistent behavioural tendencies (i.e. personality), which were shown to correlate with the individual’s propensity to learn socially [27,83–87].

For example, the observed time-lag differences between the first exposure to the first tree visit suggest these bats can retain the memory of the new information for several nights post-exposure, allowing them to weigh the costs of visiting known resources against the benefits of exploring new ones according to their current energetical requirements, which are expected to vary among individuals. This notion is further supported by the fact that almost all bats visited at least one tree prior to visiting an *F. sycomorus* after the manipulation, which implies that all bats postponed the exploration to some degree to first use more reliable resources.

Alternatively, since bats were smeared with a large amount of fruit pulp, more than expected under natural conditions, this could have prolonged the lifespan of the odour in the cave for several nights after the manipulation. In this case, some bats may have delayed their response because they required additional insurance that the information was reliable. This could have been achieved by repeatedly smelling the odour in the roost or spending more time identifying from whom it came. Indeed, laboratory-based studies on this and similar species demonstrated that fruit bats evaluate the quality of the information before choosing a novel food item [54], and exhibit consistent differences in the propensity to act as producers or scroungers when feeding together [61].

### (d) Considering alternative sources for acquiring information

Even though foraging sites away from the colony were considered an alternative hub of information for birds and bats [46,88], we found only weak evidence for the transfer of information on trees (figure 4; electronic supplementary material, figure S2), even though encounters around trees are relatively frequent (31% of dyads tracked). Although our analysis is based on many tagged bats, it may very well be that information-related interaction occurred with non-tagged bats, especially since congregations of dozens around trees are common [50,53]. We believe similar experimental manipulations we conducted for roosts, applied to trees, may elucidate if information transmission occurs, even if rare and specific to some trees. For example, smearing bats and releasing them from trees of distinctive characteristics (e.g. highly versus barely visited), and monitoring movements of other bats from the same tree before and after.

A key limitation of our manipulations is that we only introduced odours through one vector—carried on bats’ bodies and mouths—and not via other non-bat means (e.g. carried by the wind). Importantly, drawing a more conclusive interpretation necessitates examining whether odour arriving at the roost without a bat mediator would be used as an information source, suggesting it can be delivered non-socially. We believe non-bat vectors are less likely, however, mainly because the caves are far (>5 km) and secluded (figure 2; [53]). It is also less plausible that odour from the environment will create a strong-enough signal since they would be weaker than multiple bats entering the cave. To elucidate this option in the future, a similar manipulation could be designed whereby odour is introduced by spreading fruit-pulp of the unpredictably fruiting species in the cave, before the bats return to roost (making sure the scent remains fresh and strong enough to be detected) and testing the bats tree visitation patterns that follow. While non-social information sources warrant an examination, it is also possible that the rather non-overwhelming response we observed, with approximately 18% of naive bats visiting the trees, suggests a more complex social dynamic whereby the response to the odour is conditional upon naive bats evaluating the credibility of the information source by interacting or monitoring the behaviour of informed conspecifics. Here, too, continuous observations on the roosting behaviour of (tagged and individually identified) informed and naive bats could shed light on how dependent information transfer is on social dynamics.

## 5. Conclusions

We tested the ICH in a congregative fruit bat species that forage on spatially predictable, yet temporally unpredictable, resources, and showed how bats integrate personal spatial memory with public social information to track resources efficiently. Specifically, *R. aegyptiacus* use odour cues brought by conspecifics entering the cave-roost to learn about the timing of a resource emergence and cross-reference it with their personal spatial memory to pinpoint the trees’ location, without physically following informed roost-mates. As such, we provide, to our knowledge, the first empirical example of this information pathway in vertebrates, which has only been shown previously in eusocial honeybees. Additionally, our results highlight the use of using high-frequency, real-time, tracking data, such as reverse-GPS systems, together with experimental manipulations in the field, to learn about less-intuitive information transfer pathways than traditionally considered in the ICH, a topic that has been steadily gaining new insights for approximately 70 years.

## Data Availability

All materials, including the relevant data and R-code for the different analyses, are available from the Dryad database (raw bat tracks + MP4s)—[89] and Github (code and data for main analyses) [90,91]. Supplementary material is available online [92].
